# Reconsidering Open‐Ended Consent for Biospecimen and Health Record Research in the United States and Europe

**DOI:** 10.1002/eahr.60028

**Published:** 2025-07-14

**Authors:** Mark A. Rothstein, Prabha Rajasekaran, Edward S. Dove

**Affiliations:** ^1^ Director of translational bioethics at the Institute for Clinical and Translational Science at the University of California, Irvine; ^2^ Research associate at the Institute for Clinical and Translational Science at the University of California, Irvine; ^3^ Professor of law in the School of Law and Criminology at Maynooth University in Ireland

**Keywords:** biobanks, biobank‐based research, biospecimens, health records, Common Rule, informed consent, European Union (EU), health records, privacy, human research ethics, translational research

## Abstract

Translational and other modern forms of biomedical research often use stored biospecimens and the health records of individuals whose biospecimens will be used in research. As part of the enrollment process for biobank‐based research, individuals are frequently asked to provide informed consent for access to and use of their current and future health records. Although individuals might readily agree to give researchers access to their current health records, they might not realize that their future health records could contain new stigmatizing or embarrassing information. Reasonable limits on future health record disclosures in both the U.S. and Europe can address health privacy concerns without impeding research.

Consent for research was originally devised for a simple, immediate, and small‐scale model of biomedical research. Consent was singular and static, capturing the viewpoints and understandings of participants at a specific moment for a particular study. By contrast, modern methods of research, including translational research, often involve secondary research utilizing biospecimens and individual health records maintained by biobanks or health care institutions. In these settings and for this larger scale of research, traditional consent approaches have proven to be burdensome and inadequate. Several new consent approaches have been developed in recent years, including broad consent, but it is not clear how responsive these approaches are to research participants’ possible changing health status and values over their lifetime.

This essay explores why one‐time consent is inadequate to protect the long‐term autonomy and privacy interests of research participants in translational research and other forms of biobank‐enabled research. It discusses developments in the U.S. and Europe to illustrate varied approaches to balancing the interests of participants with the scientific imperatives of biobanks, longitudinal health records, and the secondary research that biobanks facilitate.

## OPEN‐ENDED CONSENT AND HEALTH POLICY

When biospecimens are collected for research purposes, including for a biobank, it is common to ask the individuals who provided the biospecimens to also provide informed consent for research use of their individually identifiable health records. Frequently, consent is obtained for the current research project as well as for future secondary research with the biospecimens and associated data stored in the biobank. From a scientific standpoint, it is extremely valuable to follow an individual's health over time to identify possible associations between findings from research with their biospecimens and the subsequent health of the individual. Nevertheless, open‐ended access to an individual's longitudinal health records raises an important concern about health privacy.

To illustrate this concern, a typical example might involve a 25‐year‐old patient who contributes a biospecimen such as a blood sample, biopsied tissue, or other biological material to a research biobank. When the biospecimen is collected, the individual is in relatively good health and does not have any particularly sensitive or stigmatizing health conditions. The individual perceives little privacy risk by also permitting researchers to have unlimited access to their current and future health records. Over time, however, the individual's health status markedly changes and their health record now includes potentially embarrassing information such as a record of mental illness, substance use, sexually transmitted infections, domestic violence, or reproductive health issues. If asked years later whether researchers can continue to have access to their health records, the individual might say no. However, research participants who agree to unlimited access to their health information at the start of a project are not usually asked to update their consent and few of them are likely to remember that years or decades earlier they authorized indefinite access to their future health records.

## CODES OF MEDICAL ETHICS AND THE WITHDRAWAL OF CONSENT

Beginning with the Nuremberg Code in 1947, international codes of ethics, statutes, and regulations provide that research participants have a right to withdraw from research at any time. The Nuremberg Code states: “During the course of the experiment the human subject should be at liberty to bring the experiment to an end if he has reached the physical or mental state where continuation of the experiment seems to him to be impossible.”^1^ Similarly, the World Medical Association Declaration of Helsinki states that “The potential participant must be informed of the right to refuse to participate in research or to withdraw consent to participate at any time without reprisal.”^2^ Likewise, an American Medical Association Ethics Opinion states: “physicians [should] also make clear that the individual may refuse to participate or may withdraw from the protocol at any time.”^3^ Of note, the World Medical Association Declaration of Taipei specifically addresses withdrawal in the context of biobank research, stating: “Individuals have the right, at any time and without reprisal, to alter their consent or to ask for their identifiable data to be withdrawn from the Health Database and their biological material to be withdrawn from a Biobank. This applies to future use of the data and biological materials.”^4^

Although these nonbinding provisions in ethics codes establish an individual's right to withdraw their consent, in practice it is an individually initiated opt‐out right. Unless periodic notice is provided by a biobank, health care institution, or researcher, few individuals are likely to remember that they consented to unlimited disclosures of their future health records or realize that they have a right to withdraw their consent (which itself may be limited if their biospecimens and data have been irreversibly deidentified). Thus, by itself, the right to withdraw consent affords insufficient protection for a participant who has a change in health status or other circumstances that alters their views on disclosure of their health information to researchers.

## BROAD CONSENT

As large scale biobank‐based research expanded in recent decades, it became increasingly burdensome for researchers to obtain informed consent from each participant for every new research use of their biospecimens and data. Broad consent is an approach that attempts to balance the interests of scientific discovery with research participants’ interests in autonomy.^5^ In 2018, the research regulation in the U.S. governing federally supported research (“Common Rule”) was revised to permit researchers to obtain broad consent for secondary research use of research participants’ identifiable private information or identifiable biospecimens.^6^

Broad consent for secondary research involves a single informed consent by research participants for future, unspecified, bona fide research uses of their identifiable biospecimens and data. For each new research use, however, an institutional review board (IRB) or research ethics committee (REC) usually reviews the researcher's application to ascertain whether disclosure is within the bounds of the participants’ consent and whether other research ethics requirements will be satisfied. Similar provisions have been widely adopted internationally despite some objections, including that expediency does not justify relaxing consent requirements^7^ and that the values of research participants can change over time and should be respected.^8^

In the European Union (EU), broad consent is regularly used for secondary research involving identifiable and deidentified biospecimens and data. It may also sometimes be deployed as the lawful basis to access and use (“process”) personal data under the EU's General Data Protection Regulation (GDPR), which is the EU's main data protection regulation. Under the GDPR, consent must be freely given, specific, informed, and unambiguous. The individual's wishes must be evidenced by a statement or clear affirmative action signifying agreement to the access and use of personal data relating to them. Language in the legislative interpretation of the GDPR in Recital 33 supports “broad consent” for scientific research activities like biobanking, although the term is not used in the legislation itself.^9^ But to process the health data of research participants, broad consent is considered lawful only if the scope of the research purposes for which the health data will be used is narrowed sufficiently to specific areas and types of research questions.

Further clarification of broad consent in the EU is provided by the European Data Protection Board, an independent EU body whose purpose is to ensure consistent application of the GDPR. According to the Board, “[Broad consent] cannot be asked and relied on for processing health data for any kind of—unspecified—future research purposes. However, the concept of broad consent could be relied on for different research projects that fall within the scope of that broad consent and that meet certain additional safeguards.”^10^

Thus, in the EU, broad consent cannot be interpreted as blanket consent. Specificity of research areas and questions are required up front, including if, as part of a study, individuals are providing consent for indefinite access to their health records.^11^ If a study obtains participants’ consent for that specific study to process their health data, and not more broadly for related research, the investigators have a legal obligation to ensure they are processing health data within the scope of the consent obtained, whether it is specific or broad.

Yet, even with the full force of the GDPR and any applicable EU member state national data protection laws, broad consent can be crafted in a way that is both in accordance with the law and also enables indefinite access to individuals’ health records so long as indefinite, future access (including for secondary use of personal data) is considered to fall within the area of research specified in the original consent. From a translational bioethics perspective, it may be difficult to modify broad consent to harmonize real‐world design challenges with the privacy interests of research participants.

## DURATION OF CONSENT

The issue of open‐ended consent arises when researchers and biobanks are permitted to obtain individually identifiable health information pursuant to a research participant's consent or authorization without

**To safeguard the privacy of research participants, the Privacy Rule should be amended to limit access to individually identifiable future health records for a specific length of time, such as five years**.

an end date. Such an arrangement is not prohibited in the U.S. by the Common Rule, which states that broad consent for research using identifiable specimens or information must state the period of time they may be used in research, but the “period of time could be indefinite.”^12^ In addition, the Privacy Rule of the Health Insurance Portability and Accountability Act (HIPAA) is more specific, requiring that authorizations include “[a]n authorization date or an expiration date that relates to the individual or the purpose of the use or disclosure. The statement ‘end of the research study,’ ‘none,’ or similar language is sufficient if the authorization is for a use or disclosure of protected health information for research, including for the creation and maintenance of a research database or research repository.”^13^ As illustrated in the previous example of the 25‐year‐old sample donor, such open‐ended health record authorizations diverge from the Common Rule's intent of supporting the autonomy of research participants and the Privacy Rule's statutory and regulatory purpose of protecting health privacy.

To safeguard the privacy of research participants, the Privacy Rule should be amended to limit access to individually identifiable future health records for a specific length of time, such as five years. Even in the absence of a legal requirement, periodic reconsenting of sample donors could be part of a biobank's operating procedures or could be required by an IRB in the consent document that establishes a biobank or in access to biospecimens and data from a biobank for a particular study. The relative ease of contacting individuals electronically to obtain additional consent makes it feasible to add a periodic notice and consent updating requirement.

Researchers are likely to have two main objections to such a change in long‐term consent. First, some individuals could be lost to follow up, making it impossible to obtain additional consent. For these individuals there would be no data loss to the research because biospecimens and previously generated health information would still be accessible. Newer health information from a missing individual would be unavailable. Second, some individuals could decline to renew their consent, thereby undermining the longitudinal utility of the biobank. Nevertheless, a fundamental principle of research ethics is that a consent document must include a statement, such as one required by the Common Rule, that “the subject may discontinue participation at any time without penalty or loss of benefits to which the subject is otherwise entitled.”^14^ Concerns about diminished scientific value or burdens on researchers are insufficient to overrule this foundational and longstanding right of participants.

In the EU, the process for researchers to obtain access to current and future health records, and the scope of that access and duration, will likely depend on compliance with a panoply of legal and regulatory requirements, including the GDPR, individual EU member state data protection laws, confidentiality and privacy laws (including the common law), freedom of information laws, biobank and medical records laws (to the extent such laws exist), the EU Data Governance Act,^15^ and the new European Health Data Space (EHDS) Regulation, which governs the use and exchange of electronic health data across the EU.^16^ For example, the new EHDS Regulation in Recital 77 states: “Given the sensitivity of electronic health data, health data users should not have unrestricted access to such data. All secondary use access to the requested electronic health data should be done through a secure processing environment.”^17^

However, despite this panoply of legal and regulatory requirements, in principle and at an EU level, there is no legal prohibition per se against indefinite access to participants’ health records, including by other researchers, provided the original consent to such access was valid, relevant authorizations or permissions were obtained (including from data controllers, who are individuals, companies, or other entities that determine the purposes and means of processing personal data), and a secure processing environment was in place.

To illustrate this conditional “green light” for indefinite access, we can look to Chapter IV of the EHDS Regulation, which governs the secondary use of electronic health data, establishing a secure and regulated framework for accessing data beyond direct patient care. Article 68(12) of the Regulation provides that “data permits” (i.e., an administration decision to process certain electronic health data) issued to “health data users” (i.e., those who have been granted lawful access to electronic health data for secondary use pursuant to a data permit) by “health data access bodies” (i.e., a body established in each EU member state allowing data users such as researchers and policy‐makers to apply for access to electronic health data) are time limited. Specifically, the provision stipulates that data permits to enable access to electronic data must be issued only for the duration necessary to fulfill the requested purposes and that duration in any event must not exceed 10 years. Although this is a step in the right direction and consonant with this essay, it is not ironclad. For example, the same provisions allow health data users to request an extension to the permit for an additional 10 years, and the permit only covers electronic health data falling within the scope of the EHDS Regulation. It is possible that other kinds of health records, including records that have not been processed in an electronic form, may be obtained without any duration limit imposed on researchers.

Separate from the EHDS Regulation provisions, it is clear that EU member states as well as other European countries may impose time restrictions on access to health records for research purposes following initial consent, but to our knowledge none have done so explicitly. For example, biobank laws in Sweden^18^ and Finland^19^ do not specify or limit the duration of consent. In the U.K., participants in the UK Biobank give explicit unlimited consent for the biobank to access all their medical and health‐related records when they first join the study. The consent form states: “I give permission for access to my medical and other health‐related records, and for long‐term storage and use of this and other information about me, for health‐related research purposes (even after my incapacity or death).”^20^ This provision is applicable only if the initial consent was informed.

Beyond legal compliance, ethical practice dictates that researchers update participants regularly as to research being done with their biospecimens and data and at least provide the opportunity to opt out of future health record disclosures. In this way, researchers better respect participants by ensuring that their consent to health record disclosure remains valid on an ongoing basis. Furthermore, of perhaps even greater significance, the UK Biobank only collects coded data about health conditions—another safeguard and good practice to keep ongoing data collection and access limited to what is necessary to fulfill the research aims.

In our view, the UK Biobank system of collecting only coded data is a step in the right direction to sustain the minimum requirements of a translational bioethics approach to longitudinal data access and utilization. Yet even in this example, challenges remain: the added step of securing indefinite access to records in this case is the permission from the data controllers of those records, the participants’ primary care physicians. To date, only a minority of primary care physicians have approved the release of health records data to the UK Biobank, most likely due to privacy and security concerns.^21^ Although some individuals (and data controllers) might still have legitimate privacy and security concerns about the research use of even their deidentified sensitive information, the balancing of personal privacy and autonomy with the scientific imperative seems to us a more reasonable approach, especially if linked with a transparent informed consent process.

## DEIDENTIFICATION OF HEALTH INFORMATION

Deidentified biospecimens and health records may have less scientific value than fully identifiable sources because, among other reasons, deidentification prevents more detailed linkage or follow‐up. But for some or many researchers, providing enhanced privacy protections for participants as well as reducing researchers’ perceived burdens of legal or administrative requirements are the impetus for deidentification.

In the U.S., researchers are permitted to deidentify biospecimens and health records and then use them in research without any notice to or consent from the individual or any external oversight.^22^ Notwithstanding the scientific and ethical concerns raised by this process, because the option is available, researchers in the U.S. may decline to use broad consent or other measures with consent requirements.^23^

As noted above, the UK Biobank only collects coded data about health conditions. Letters and notes of conversations between a participant and their physician would not be shared with the UK Biobank. In addition, all data about participants are held separately from their personal identifying information (such as name and address) in encrypted form for optimum protection. From a legal and ethical perspective, this is more advisable than holding all identifiable data about participants in the same dataset.

For EU countries, under the EHDS Regulation, any researcher access to electronic health data under this Regulation is presumed to be made available in an anonymized format. If a researcher has sufficiently demonstrated that the purpose of processing cannot be achieved with anonymized data, then electronic health data may be provided in pseudonymized (i.e., key‐coded) format, with the “key” not being in the possession of the researcher.^24^ The researcher in this case would also need to establish a legal basis under Articles 6(1) and 9(2) of the GDPR to receive access to other than anonymized electronic health data for secondary use.

## SEGMENTATION OF ELECTRONIC HEALTH RECORDS

Another approach to preventing the disclosure of sensitive information from electronic health records (EHRs) is to permit all patients to segment a portion of their health information in a limited number of predetermined categories, such as mental health and reproductive health.^25^ As proposed in the U.S., researchers as well as clinicians seeking to obtain health information in these categories would be required to obtain additional consent from a patient or research participant to access segmented files. An emergency feature could grant broader access in special situations. Clinical decision support could scan the entire record to assess whether there is, for example, a possible drug interaction involving medication prescribed for a condition in the segmented category. Although technologically feasible, in the U.S., segmentation has not received much support.

Segmentation of health records would also appear to be possible in EU member states, including under the new EHDS Regulation. Recital 17 of the EHDS Regulation spells out the rationale for segmentation: “[People] might not want to allow access to some parts of their personal electronic health data while enabling access to other parts. This could especially be relevant in cases of sensitive health issues such as those related to mental or sexual health, sensitive procedures such as abortions, or data on specific medication which could reveal other sensitive issues. Such selective sharing of personal electronic health data should therefore be supported and implemented … .”^26^

However, while segmentation is possible under EHDS Regulation Articles 5 and 8, these provisions only govern access to records by health professionals and health care providers (i.e., for direct care purposes; this is known as “primary use”). Regarding secondary use of health records for research purposes, under Article 71, individuals can opt out of whether they want their personal electronic health data to be processed for secondary use (this right to opt out is in turn subject to exceptions under the provision). In other words, the Regulation does not indicate that segmentation is possible for secondary use access. As noted in the section above, to preclude the identification of individuals, secondary use of electronic health data is based on pseudonymized or anonymized data. However, it does not appear that individuals can predetermine which categories of health data within their EHRs they want to make available to researchers; rather, it appears to be an all‐or‐nothing approach.

Segmentation represents a trade‐off between a patient's right to control their health information and the status quo of all‐inclusive health information availability. Segmentation is technically feasible through EHR design modifications, and even though it would involve some additional costs and inconvenience, it would likely provide significant privacy benefits.^27^ In research settings, access to health records would depend on the types of segmented categories, patient decisions to segment, and the scope of consent for research.

## ACCESS TO AND CORRECTION OF HEALTH RECORDS

In the U.S., under the HIPAA Privacy Rule, individuals have a right of access to their health records,^28^ and they may request that a covered entity (e.g., health care provider) correct errors, but the covered entity need not grant such a request.^29^ As a practical matter, there are few requests to correct health records and very few requests from individuals are granted.

In Europe, under data protection law and subject to possible conditions, a person is entitled to find out if there is information about them held in a medical record, to access those records, and to have the records corrected if they are inaccurate. This applies to both public and private health care providers. The overarching principles of data protection—that data must be accurate and up‐to‐date (among other principles)—are established by the GDPR and other data protection‐related laws in the individual EU member states.^30^

## DESTRUCTION OF HEALTH RECORDS

Another limitation on researchers’ ability to access participants’ health records are laws and policies on the destruction of health records. In the U.S., federal (e.g., HIPAA, Medicare) and state laws require retention rather than destruction. Physicians are generally required to retain health records for between five and seven years. Mandatory retention times for hospitals are often longer, and in some states, records must be retained at least for 10 years.^31^ Research institutions with biobanks usually have policies of unlimited retention that are facilitated by EHR systems and no need to store paper records.

In some European countries, even if a research participant consents to indefinite access to their health records, from a practical standpoint, such records may not be available indefinitely. For example, in Ireland, pathology clinical records may be retained between one and 30 years, depending on the record type, while a deceased person's health records are destroyed eight years after death. There are different retention periods depending on the individual and their status, as well as the type of health information recorded.^32^

## CONCLUSION

Informed consent was conceptualized and initially adopted when biomedical research consisted largely of short‐duration, one‐time studies with separate consent for each study. In that context, an individual's paper medical records were dispersed among multiple health care providers. Today, longitudinal, comprehensive, and interoperable EHRs, combined with scientific breakthroughs in computer technology and internationally collaborative biomedical research have generated a previously unimaginable scale of studies, participants, biospecimens, health records, data sets, and duration. A significant, yet largely unaddressed health privacy issue arises from biobanks and researchers using open‐ended consent to access participants’ future health records. In light of the expansive nature of modern biomedical research, including translational research, additional health privacy protections should be adopted by legislation, regulation, research ethics policies, or research institution best practices to set initial time limits on the ability to access and use future health records and to require periodic notification and reconsenting of participants.

The current practice of using open‐ended consent for indefinite disclosure of future health records risks exploiting research participants by placing the burden on them to remember the terms of their past generous donations to scientific research and to opt out if they want to withdraw from continued participation. As paragraph 9 of the Declaration of Helsinki states: “The responsibility for the protection of research participants must always rest with physicians or other researchers and never with the research participants, even though they have given consent.”^33^

Translational bioethics focuses on applying broad bioethics perspectives to modern translational research methods.^34^ The use of open‐ended consent for biospecimen and health record research is inconsistent with such an approach. Therefore, consent approaches and policies should be reconsidered and redesigned to advance the significant individual and societal interests in translational research.
